# The Role of Hydrogen Sulfide in the Regulation of the Pulmonary Vasculature in Health and Disease

**DOI:** 10.3390/antiox14030341

**Published:** 2025-03-14

**Authors:** Philip I. Aaronson

**Affiliations:** Department of Inflammation Biology, School of Immunology and Microbial Sciences, Faculty of Life Sciences and Medicine, King’s College London, London SE1 9RT, UK; philip.aaronson@kcl.ac.uk

**Keywords:** hydrogen sulfide, reactive sulfur species, pulmonary hypertension, hypoxic pulmonary vasoconstriction, O_2_ sensing, NFκB, Nrf2, monocrotaline, arterial remodeling

## Abstract

The gasotransmitter hydrogen sulfide (H_2_S; also termed sulfide) generally acts as a vasodilator in the systemic vasculature but causes a paradoxical constriction of pulmonary arteries (PAs). In light of evidence that a fall in the partial pressure in oxygen (pO_2_) increases cellular sulfide levels, it was proposed that a rise in sulfide in pulmonary artery smooth muscle cells (PASMCs) is responsible for hypoxic pulmonary vasoconstriction, the contraction of PAs which develops rapidly in lung regions undergoing alveolar hypoxia. In contrast, pulmonary hypertension (PH), a sustained elevation of pulmonary artery pressure (PAP) which can develop in the presence of a diverse array of pathological stimuli, including chronic hypoxia, is associated with a decrease in the expression of sulfide -producing enzymes in PASMCs and a corresponding fall in sulfide production by the lung. Evidence that PAP in animal models of PH can be lowered by administration of exogenous sulfide has led to an interest in using sulfide-donating agents for treating this condition in humans. Notably, intracellular H_2_S exists in equilibrium with other sulfur-containing species such as polysulfides and persulfides, and it is these reactive sulfur species which are thought to mediate most of its effects on cells through persulfidation of cysteine thiols on proteins, leading to changes in function in a manner similar to thiol oxidation by reactive oxygen species. This review sets out what is currently known about the mechanisms by which H_2_S and related sulfur species exert their actions on pulmonary vascular tone, both acutely and chronically, and discusses the potential of sulfide-releasing drugs as treatments for the different types of PH which arise in humans.

## 1. Introduction

The gasotransmitter hydrogen sulfide (H_2_S, here referred to as sulfide to encompass the species H_2_S and HS^−^ which co-exist in a ratio of ~45 to 55% at an intracellular pH of 7.1) has multiple effects on the cardiovascular system, including vasodilatation, cardioprotection, and angiogenesis. It also has antioxidant and inflammatory properties and inhibits extracellular matrix remodeling and vascular smooth muscle cell proliferation [1,2,3].

Hydrogen sulfide has been implicated in both the physiology and pathophysiology of the pulmonary vasculature. With regard to the former, the nature of the O_2_ sensing mechanism for initiating hypoxic pulmonary vasoconstriction (HPV), the unique rapid homeostatic vasoconstricting response of the pulmonary vasculature which functions to maintain the ventilation: perfusion ratio in the face of localized alveolar hypoxia, has been a source of controversy for the past three decades. Kenneth Olson and colleagues proposed in 2006 [4] that an increase in the intracellular sulfide concentration in pulmonary artery smooth muscle cells (PASMCs) constitutes the O_2_ sensor responsible for triggering HPV. With regard to sulfide and pulmonary pathophysiology, Chao-shu Tang, Jin-bao Du, and colleagues [5] presented evidence in 2003 that production of sulfide by the lung was diminished in a rat model of pulmonary hypertension (PH), a pathological sustained rise in pulmonary artery pressure (PAP). Moreover, sulfide supplementation in the form of a daily injection of NaHS during the hypoxic induction protocol significantly ameliorated PH and the pulmonary arterial remodeling which contributes to its development. The role of sulfide in PH has subsequently been explored in more depth through investigations employing in vivo animal models, in vitro studies on human cells, and measurements of plasma sulfide concentrations in patients with PH. An excellent and authoritative review of the evidence for the involvement of sulfide in both HPV and PH was published in 2021 by Roubenne et al. [6]. Although covering much the same ground, the present review provides an update of developments in these fields since then, and also examines several relevant older papers which were not discussed by Roubenne and colleagues. I also present an in-depth and critical analysis of the evidence showing that pulmonary production of sulfide is decreased and discuss the mechanisms which may account for this phenomenon, which was described briefly but not focused upon in the previous review.

## 2. Hydrogen Sulfide Metabolism and Signaling

**Figure 1:**
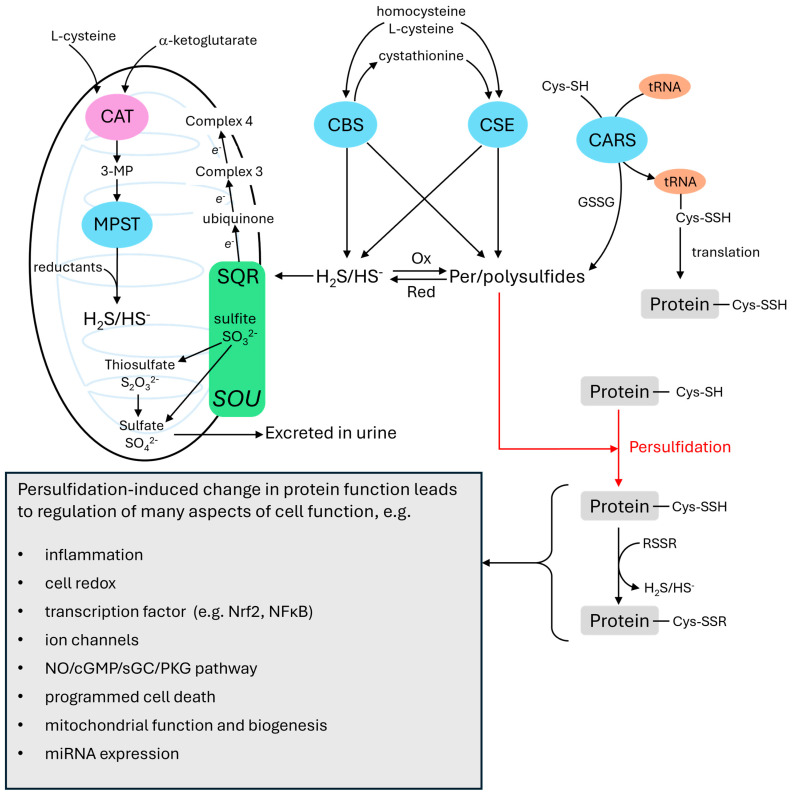
A simplified summary of sulfide and per-/poly sulfide metabolism and signaling. Sulfide and per-/polysulfides, also termed *reactive sulfur species* or *supersulfides*, are synthesized by four main enzymes. Cystathionine β-synthase (CBS) can generate sulfide from homocysteine, and sulfide from L-cysteine, whereas cystathionine-γ lyase (CSE) synthesizes cysteine from cystathionine, and uses it and homocysteine to generate sulfide. 3-mercaptopyruvate sulfur transferase (MPST) uses 3-mercaptopyruvate (3-MP) generated by cysteine aminotransferase (CAT) from L-cysteine and α-ketoglutarate to generate sulfide and pyruvate, with a reductant such as thioredoxin required to release sulfide from MPST. Cysteinyl–tRNA synthetase (CARS) uses CysS-(S)_n_-H (cysteine persulfides with one or more sulfane sulfurs) to persulfidate tRNA, generating CysS-(S)_n_-H -bound tRNA and leading to incorporation of persulfidated cysteine residues into newly-formed proteins. CARS can also form CysS-(S)_n_-H itself. Cellular sulfide and per-/polysulfides are interconvertible and exist in an equilibrium which is determined by factors including the local redox potential, the partial pressure of oxygen (pO_2_), and the activities of enzymes such as superoxide dismutase 1 (SOD1) which can oxidize sulfide to polysulfides. Once formed, sulfide is metabolized largely by the sulfide oxidation unit (SOU) in the mitochondria, as described in the text. Sulfide and (probably more importantly) per-/polysulfides can persulfide various proteins, affecting their functions both directly and via their interactions with other proteins.

Cellular labile sulfur (i.e., that which is not present in stable compounds such as methionine and cysteine and can be released as inorganic sulfur by reduction or acid treatment) [7] exists in three forms [8,9]. These are: 1. free sulfide (H_2_S/HS^−^), 2. bound sulfane sulfur, where ‘bound’ refers to a form of sulfur which is released as H_2_S when reduced, and ‘sulfane’ refers to a sulfur atom bound to two other sulfurs or one sulfur and an ionizable hydrogen, and 3. acid labile sulfur, which can be released by acid treatment and is mainly bound in iron-sulfur proteins of the mitochondrial electron transport system. Figure 1 summarizes aspects of the cellular metabolism and effects of sulfide relevant to this review (see [10,11,12,13,14] for more details).

The key point is that cellular sulfide in its free and bound sulfane sulfur forms can persulfidate cysteine resides, thereby causing changes in protein function. This is seen as the main mechanism by which sulfide exerts its signaling function in cells. In addition, because the persulfidation of cysteine residues is reversible, it has been proposed that it may also function to protect cysteine residues from (irreversible) peroxidation, which may occur during oxidative stress [9].

The free sulfide concentration in plasma and most types of tissue is thought to be in the nM range [12,15,16]. Notably, however, Levitt et al. 2011 [16] reported that the free sulfide concentration was 20–200 fold higher (a mean of ~1 μm/kg) in the aorta of the mouse, although it is not known whether this is also the case in other arteries or species (see Section 5.3 for a discussion of sulfide levels in the lung). Bound sulfane sulfur comprises a diverse set of chemical species containing chains of 2–7 sulfur atoms. These include hydrogen persulfides (HSS_n_H, which can be formed by oxidation of sulfide), persulfides (RSS_n_H/RSS_n_^−^), and polysulfides (RSS_n_R), where R can be hydrogen, cysteine, glutathione (GSH), or a protein thiol. Per- and polysulfide concentrations in cells are thought to be in the micromolar range [12,14,17]. Akaike and colleagues have recently proposed that the umbrella term ‘supersulfides’ be used to describe RSSH species and polysulfides with catenated (sulfane) sulfur atoms (RSS*_n_*R), including those formed by the persulfidation of cysteine residues in proteins. Another widely used term is ‘reactive sulfur species’ (RSS) [12,13].

Sulfide can be synthesized by at least six enzymes found in eukaryotic cells. The most important of these are cystathionine-γ-lyase (CSE), and cystathionine-β-synthase (CBS), which are located in the cytoplasm, and 3-mercaptopyruvate sulfur transferase (MPST), which is mainly in the mitochondria. Under hypoxic conditions, CSE translocates to the mitochondria [18] and CBS levels in the mitochondria also rise as its breakdown by the mitochondrial enzyme Lon protease is inhibited [19].

All three of these enzymes are present in cardiovascular cells [20]. Nevertheless, CSE, which synthesizes sulfide mainly from L-cysteine and homocysteine using pyridoxal 5′-phosphate (vitamin B_6_) as a cofactor, is often described as the most important sulfide-producing enzyme in the cardiovascular system, and has drawn most attention with regard to the effects of sulfide in pulmonary arteries (PAs). The presence of CSE mRNA and/or protein has been detected in lung tissue from mice, rats, sea lions, and cows [5,21,22], rat and sea lion PAs [21,22,23], mouse, rat, and cow PASMCs [21,22,24], and pulmonary artery endothelial cells (PAECs) from mice [24], but not cows [21]. CBS, which also uses pyridoxal 5′-phosphate as a cofactor, is present in bovine PAECs but not PASMCs [21]. Sun et al. (2011) [25] and Luo et al. (2013) [26] detected CBS protein in rat PA, whereas Madden et al. (2012) [27] and Prieto-Lloret et al. (2015) [28] failed to find CBS protein in rat lungs and CBS mRNA in rat PAs, respectively.

The presence of CBS in mouse lungs is similarly disputed [24,29]. MPST, which is expressed in rat PAs and cow PAECs [23,27], utilizes α-ketoglutarate and 3-mercaptopyruvate (3-MP) to synthesize sulfide. The reaction involves the transfer of a sulfur atom from 3-MP to MPST and the subsequent release of sulfide from the enzyme by a reducing agent such as thioredoxin or dihydrolipoic acid (DHLA). 3-MP is generated from cysteine by the enzyme cysteine amino transferase (CAT) (also called aspartate aminotransferase), which can alternatively synthesize oxaloacetate from a-ketoglutarate and aspartate.

Per- and polysulfides are synthesized by mainly by sulfurtransferases, enzymes which, as their name suggests, transfer sulfane sulfurs between molecules. The most important sulfurtransferases are thought to be cysteinyl tRNA synthetases (CARSs), which synthesize CysSSH and can incorporate it into proteins during translation [30]. There is recent evidence that the production of supersulfides by CARS2 exerts a protective action on the airways in chronic lung disease [31]. The sulfide-producing enzymes CSE, CBS, and MPST also synthesize per-and polysulfides. Thiosulfate (SSO_3_^2−^), which contains a sulfane sulfur, is formed during the oxidation of sulfide in the mitochondria. Bound sulfane sulfur can be released as sulfide by reductants such as GSH, DHLA and thioredoxin, implying that a reducing shift in the cell redox potential could raise the cellular [sulfide]. On the other hand, sulfide can be oxidized to persulfide by H_2_O_2_ [32], and also by superoxide dismutase 1 (SOD1) [33].

Importantly, both H_2_S and per-/polysulfides can persulfidate (sulfurate) protein thiols, and it has been argued that per-/polysulfides are more important than sulfide in this respect. One reason for this is that per-/polysulfides are much more abundant in cells. In addition, whereas sulfide can react with protein thiols only when it, or the thiol, is in a oxidized form, this is not required for per-/polysulfide-mediated thiol persulfidation [8]. Sulfurtransferases such as CARS and MPST can also directly persulfidate protein thiols [30,34]. As with other oxidative cysteine modifications important in signaling, persulfidation can be reversed by nicotinamide adenine dinucleotide phosphate oxidase (NADPH)-dependent reducing systems such as thioredoxin/thioredoxin reductase [9].

The catabolism of sulfide is thought to be mediated mainly by the mitochondrial ‘sulfide oxidation unit’ (SOU). The first step in this process is mediated by the reaction of sulfide with the enzyme sulfide:quinone oxidoreductase (SQR), which results in the sulfidation of GSH to glutathione disulfide (GSSG) and the transfer of two electrons to co-enzyme Q and thence into the electron transport chain (ETC). GSSG can then be oxidized through reactions catalyzed by persulfide dioxygenase (ETHE1), rhodanese (thiosulfate sulfurtransferase) and sulfite oxidase (SUOX), ultimately resulting in the formation of thiosulfate or sulfate (SO_4_^2−^) [35] which is excreted in the urine. As with per-and polysulfides, reduction of thiosulfate can give rise to sulfide, and it has been proposed that this occurs in the mitochondria under hypoxic conditions [36].

Although its role as a signaling species has increasingly been overshadowed by that of supersulfides, there is recent evidence that by feeding electrons into the mitochondrial coenzyme Q pool via its reaction with SQR, sulfide at concentrations high enough to block complex 4 may reverse the reaction by which succinate is oxidized to fumarate at complex 2 [37]. Since this would alter the equilibria of reactions within the Krebs cycle, it may provide a mechanism by which sulfide itself could regulate cell function by controlling the flux of intermediates through cellular metabolic pathways [38].

## 3. Hydrogen Sulfide as an O_2_ Sensor

The concept that the metabolism of sulfide can act as an acute (i.e., fast-responding) O_2_ sensor, introduced by Kenneth Olson and colleagues in 2006 [4,21,27,36,39,40,41] is based on the premise that cellular free [sulfide], which is thought to be very low under basal conditions, increases rapidly during hypoxia in proportion to the fall in the partial pressure of oxygen (pO_2_). This was proposed to occur because the catabolism of sulfide depends almost entirely on its oxidation by the SOU, which would be expected to diminish as the pO_2_ falls. In contrast, the synthesis of sulfide, which occurs via both enzymatic and non-enzymatic pathways, persists under hypoxic conditions [42]. Furthermore, reduction of the mitochondrial matrix, which has been shown to occur in PASMCs during hypoxia [43] was proposed to cause the release of sulfide from thiosulfate (S_2_O_3_^2−^), which is produced in the mitochondria during the catabolism of sulfide [36].

The strongest evidence that an increase in the cellular sulfide concentration contributes to O_2_ sensing has come from studies of chemoreceptor cells, which monitor the pO_2_ level in arterial blood and initiate compensatory homeostatic cardiovascular and respiratory responses to hypoxia (n.b. arterial chemoreceptors also orchestrate responses to a range of other perturbations in the chemical composition of the plasma). Evidence for a role of sulfide in O_2_ sensing was first reported in trout gills, the first pair of which serve as O_2_ sensors in this species and are homologous to the mammalian carotid body (CB) [40]. Evidence then emerged that a rise in sulfide was also an O_2_ sensor in the mammalian CB [44,45,46,47,48,49], although it was suggested that hypoxia was increasing sulfide, not by inhibiting its metabolism, but by decreasing carbon monoxide (CO) production by hemoxygenase-1 (HO-1) [47]. This was shown to inhibit protein kinase G (PKG)-mediated phosphorylation of CSE, resulting in its activation [49]. The concept that sulfide is involved in O_2_ sensing in the CB was challenged by evidence that CSE knockout did not affect acute O_2_ sensing in the CB [50], and that pharmacological blockers of CSE and CBS did not prevent hypoxia-induced inhibition of the TWIK-related acid-sensitive K^+^ (TASK) current or the rise in [Ca^2+^]_i_ which play important roles in O_2_ sensing in CB chemoreceptor cells [51]. However, it has recently been reported [52] that sulfide contributes to O_2_ sensing in mouse CB via the persulfidation of olfactory receptor 78, which results in Ca^2+^ influx mediated by cyclic nucleotide-gated channel α2.

### 3.1. Sulfide and HPV

HPV is a rapidly-developing constrictor response of the pulmonary vasculature, particularly at the level of its small muscular precapillary arteries, to alveolar hypoxia. There is evidence that multiple effector mechanisms within PASMCs, including (but not limited to) voltage-gated K^+^ (K_V_) and TASK channel inhibition, opening of voltage-gated Ca^2+^—channels and transient receptor potential channels, Ca^2+^ release from the sarcoplasmic reticulum via ryanodine and inositol tris phosphate receptors, store-operated Ca^2+^ influx, and RhoA/Rho kinase mediated Ca^2+^ sensitization, contribute to the constriction. However, the nature of the O_2_ sensor(s) which engage(s) these mechanisms remains controversial [53].

Olson and colleagues (Olson et al., 2006) [4] proposed that the O_2_ sensor for HPV is a rise in the intracellular sulfide concentration which, as described above, is caused by a hypoxia-induced fall in sulfide metabolism. This concept was supported by observations that application of sulfide (as NaHS) consistently mimicked the effects of hypoxia on vascular tone in more than a dozen different types of blood vessels from diverse species [41,54]. Notably, both NaHS and hypoxia caused a biphasic contraction of rat PA. Also sulfide ‘donors’ such as cysteine and glutathione were shown to enhance HPV [27,41]. More direct evidence for the involvement of sulfide in HPV came from observations that antagonists of sulfide-synthesizing enzymes attenuated HPV in rat and bovine PAs [4] and perfused rat lungs [27] and that the reducing agents DHLA and dithiolthreitol, which would be predicted to release sulfide from thiosulfate in the mitochondria, increased the amplitude of HPV [36]. On the other hand, in a more detailed study, Prieto-Lloret et [28] saw no effect of inhibitors of CSE (propargylglycine) or CAT/MPST (aspartate) on HPV in rat intrapulmonary arteries. Moreover, 1 × 10^−3^ mol/L dithiolthreitol pretreatment abolished rather than enhanced HPV (as also observed by Du et al., 2005 [55]), and whereas 1 × 10^−3^ mol/L L-cysteine increased the amplitude of HPV as shown by Olson et al., 2010 [41], it had a similar effect on the contraction to prostaglandin F_2α_ under normoxic conditions.

The possibility that hypoxia increases cellular [sulfide] quickly enough to initiate HPV, which develops within seconds to minutes, received circumstantial support from experiments showing that sulfide was produced rapidly under anoxic conditions by sea lion and cow lung homogenates provided with the necessary cofactors and substrates required for CSE, CBS and MPST, and then was promptly consumed when O_2_ was admitted to the reaction chamber [21]. Similar results were reported in trout gill homogenates and cardiac mitochondria [40,56]. The rate of sulfide consumption by a suspension of bovine PASMCs had a hyperbolic dependence on the oxygen concentration, with half-maximal H_2_S consumption observed at ~6 mmHg, and the curve describing this relationship had a pO_2_ dependency which was very similar to that of HPV recorded in bovine PAs [21].

Studies in several other types of cells also indicate that hypoxia can cause a large increase in sulfide within minutes. Peng et al. (2010) [47] reported that 60 min of moderate hypoxia (pO_2_ ~39 mmHg) increased cellular [sulfide] levels approximately three-fold in mouse CB homogenates, and the same laboratory has recently shown that this level of hypoxia increased the persulfidation of proteins in CB sections within 5 min [52]. Krause et al. [57], using the sulfide indicator, 7-azido-3-methylcoumarin (AZMC), reported a significant increase in sulfide production by pig tracheal epithelium subjected to severe hypoxia (pO_2_ ~2 mmHg) after 2 h. Hypoxia (pO_2_ ~8 mmHg) also caused a small increase in sulfide production measured over 60 min in erythrocytes [58] However, the only comparison of sulfide production under normoxic vs. hypoxic conditions which has been carried out in PASMCs, which also used AZMC, showed that its rate of production over 5 h was the same when the cells were incubated in PSS equilibrated with 5 and 21% O_2_. Nevertheless, more detailed investigations using shorter incubation times, a range of levels of hypoxia, and measurements of protein persulfidation, is clearly required before any meaningful conclusions about the effects of hypoxia on sulfide levels in PASMCs can be drawn.

### 3.2. Per-/Polysulfides and HPV

As it has become increasingly apparent that sulfide exists in equilibrium with per- and polysulfides which may have a primary role in protein persulfidation and therefore regulation of cell function [11,42,59], it has recently been suggested that it is the accumulation of these species rather than sulfide per se which constitutes the important O_2_ sensing signal [42]. However, as of yet no studies directly evaluating the involvement of either per-/polysulfides or protein persulfidation in HPV have been published. Also, Olson et al. (2020) [60] found that polysulfide production by bovine PASMCs, which they measured using 3′,6′-Di(O-thiosalicyl)fluorescein (SSP4), was unchanged during the first 5 h of hypoxia (5% O_2_) compared to 21% O_2_. In contrast, per-/polysulfide production was increased over 20 h of hypoxia, leading them to suggest that sulfide or per-/polysulfides might be involved in long term O_2_ sensing. Whilst these results do not support a role for reactive sulfide species in HPV, more thorough investigations are warranted, especially in light of evidence that hypoxia induces a marked increase in per- and polysulfides in mouse aortic endothelial cells within 30 min through a mechanism involving the phosphorylation of CSE by AMP kinase [61].

### 3.3. Mechanisms of Sulfide-Induced Contraction

Although sulfide has been shown to act as a vasodilator in the majority of studies examining its effects on vascular tone, it can also cause constriction in some blood vessels, most notably pulmonary and cerebral arteries [4,62,63,64]. The effect of sulfide on vascular tone in PAs is complex, indicating that it evokes both constricting and dilating mechanisms in a time- and concentration manner. For example, in bovine PA, when applied on top of a contraction induced by 10^−7^ M U46619, NaHS caused a relaxation at lower concentrations (10^−8^–10^−5^ mol/L) and a contraction at higher concentrations; a similar pattern occurs in duck PAs [54]. In contrast, an opposite pattern of concentration-dependency was observed in phenylephrine-preconstricted mouse aorta and carotid artery, in which applying NaHS at low concentrations (10^−8^–3 × 10^−6^ mol/L) caused contraction, whereas relaxation occurred at higher concentrations (3 and 10 × 10^−5^ mol/L) [65].

10^−3^ mol/L NaHS causes a triphasic response (small transient contraction, relaxation, sustained contraction) in preconstricted rat PA, whereas a monophasic sustained contraction is observed at 1 and 3 × 10^−5^ mol/L [23,54]. In human PAs and perfused lung, however, NaHS was reported to cause no effect on vascular tone at 2 × 10^−5^ mol/L and a dose-dependent relaxation at concentrations of 5 × 10^−5^ mol/L and above [66].

The best-characterized mechanism by which sulfide causes vasodilation is an activation of the nitric oxide (NO)/soluble guanylate cyclase (sGC)/cyclic GMP/PKG axis, which occurs at multiple points in this pathway [67] including the persulfidation of PKGα1 [59], which is the predominant form of PKG in the vasculature. PKG activation exerts a powerful vasodilating influence via numerous mechanisms including Ca^2+^ desensitization, opening of K^+^ channels, and inhibition of inositol tris phosphate (IP_3_) -induced Ca^2+^ release. Sulfide also exerts a vasodilating effect in PAs and other types of arteries by directly stimulating the opening of K_ATP_ channels. It is thought to do so by persulfidating the sulfonylurea receptor (SUR) 2B subunit of the channel [68], which is expressed in PAs [25,69]. Further potential vasodilating mechanisms include inhibition of the RhoA/Rho kinase pathway [70], store-operated Ca^2+^ entry [71] and voltage-gated Ca^2+^ channels [72] as well as activation of K_V_ channels [73,74].

The contractile effects of sulfide, especially in PA, are less well understood. However, there is evidence in systemic arteries that a fall in the arterial cyclic AMP content is involved [64,75], and also that sulfide-induced contraction is endothelium- and NO-dependent (Kubo et al., 2007) [76]. In an elegant study which provided a potential explanation for both observations, Mitidieri et al., 2021 [65] demonstrated that low concentrations of sulfide evoked a contraction of phenylephrine pre-constricted mouse aorta and carotid arteries which was endothelium, NO, and sGC—dependent. This contraction was due to a sulfide-induced stimulation of phosphodiesterase, which was lowering the levels of the vasodilating second messengers cyclic GMP and cyclic AMP, but was not affecting the concentration of inosine 3′,5′-cyclic monophosphate (cyclic IMP), a vasoconstricting cyclic nucleotide which is produced by sGC along with cyclic GMP. They also showed that contractions evoked in these arteries by phenylephrine and several other stimuli were smaller in CSE knockout mice, implying that even endogenous levels of sulfide act to non-specifically promote vasoconstriction by evoking a similar ‘biasing’ of cyclic nucleotide levels. The production of cyclic IMP in an endothelium/NO/sGC-dependent manner has also been implicated in the hypoxia-induced of porcine coronary arteries [77,78], in which it has been ascribed the activation of NAD(P)H:quinone oxidoreductase [79] and/or the inhibition of phosphodiesterases 1 and 5 [80]. Nevertheless, the NO/sGC dependency of this mechanism would appear to rule out its involvement in HPV, which is potentiated rather than suppressed in endothelial NO synthase (eNOS) knockout mice and in the presence of pharmacological blockers of eNOS and sGC [81,82,83].

Koenitzer et al. 2007 [84] made the interesting observation that sulfide caused a small contraction when applied to phenylephrine-pre-constricted rat aorta at a pO_2_ of 114 mmHg, whereas at a pO_2_ of 23 mmHg it caused a profound vasodilation. They speculated that the contraction may have been caused by an oxidation product of sulfide, and that PAs might contract to sulfide because they exist in a more O_2_-rich milieu than do systemic arteries, which typically relax to sulfide. Nevertheless, reports of sulfide-induced vasorelaxation of systemic arteries under hyperoxic conditions [85,86] argue against this concept.

Prieto-Lloret and colleagues [23,87] investigated the mechanism of the sulfide-induced contraction in rat small PAs using NaHS. The arteries were slightly pre-constricted using prostaglandin F_2α_ in order to magnify the sulfide contraction. Lower concentrations of NaHS (1 and 3 × 10^−5^ mol/L) caused a small sustained contraction associated with a hyperpolarization of the mitochondrial membrane potential (Ψ), whereas higher concentrations of NaHS (10^−4^ to × 10^−3^ mol/L) caused a triphasic contractile response similar to that previously reported [4], in which a transient rise in tension was followed by a relaxation and then a second larger, and more sustained, contraction. Both contractions coincided with mitochondrial hyperpolarization, and mitochondrial depolarization occurred during the relaxation. The sustained contraction to 10^−3^ mol/L sulfide was inhibited by antagonists of protein kinase C (Gö6983), ryanodine receptors (5 × 10^−5^ mol/L ryanodine), NADPH oxidase (VAS2870), and rho kinase (Y27632), as well as by the complex 3 blocker myxothiazol and the antioxidant TEMPOL, but was insensitive to block of voltage gated Ca^2+^ channels (nifedipine) and complex 1 (rotenone). Similarly, contractions evoked by 3 × 10^−5^ mol/L sulfide and the sulfide precursor cysteine (1 × 10^−5^–3 × 10^−3^ mol/L) were antagonized by Gö6983 and VAS2870. It was also found that application of 10^−3^ mol/L sulfide to cultured PASMCs led to an increase in the production of reactive oxygen species which was greatly diminished in cells in which SQR expression had been knocked down with siRNA. Based on these and other results, the authors concluded that the contraction to sulfide was due to its metabolism by SQR, which, by feeding electrons into the co-enzyme Q pool of the ETC, was causing an increased production of H_2_O_2_ by complex 3. This possibility is in line with evidence that sulfide also increased reactive oxygen species (ROS) production in trout gill [88], and with the proposal by Schumacker and colleagues that HPV is triggered by complex 3-mediated ROS production (Waypa et al., 2001) [89].

Intriguingly, the sensitivity of the sustained sulfide contraction to the pharmacological agents used in this study overlaps in many respects with that of phase 2 (sustained) HPV, which was also shown to be insensitive to nifedipine but blocked by Gö6983, ryanodine, Y27632, myxothiazol, and TEMPOL [90,91,92]. On the other hand, HPV was not blocked by VAS2870 [90]. However, based on the close resemblance between the mechanisms of hypoxia and sulfide in causing PA contraction, one might speculate that a rise in the mitochondrial sulfide concentration could contribute to triggering HPV by enhancing ROS production at complex 3. Nevertheless, there is no evidence that hypoxia causes a rapid increase in mitochondrial sulfide levels in PA, and the question of why applying exogenous sulfide tends to have a more prominent constricting effect in PAs compared to systemic arteries remains unanswered.

## 4. Pulmonary Hypertension

### 4.1. Classification of Pulmonary Hypertension

PH comprises a heterogeneous group of conditions in which the mean PA pressure is elevated to ≥20 mmHg (from its normal level of ~14 mmHg) due to an increased pulmonary vascular resistance (PVR). This causes hypertrophy and eventually failure of the right ventricle, leading to premature mortality.

PH is classified into five categories, each which has its own clinical, functional and pathological properties [93]. The archetypical form of PH, which is relatively rare, is referred to as pulmonary arterial hypertension (PAH, Group 1 PH) and is defined by a unique form of remodeling of the pre-capillary pulmonary vasculature which is distinguished by the presence of complex (‘plexiform’) lesions and areas of laminar concentric intimal fibrosis, both of which develop just distal to arterial branch points and cause intralumenal obstruction. In addition, pulmonary arteries exhibit medial hypertrophy and intimal and adventitial proliferation, and pulmonary arterioles, which normally contain little smooth muscle, undergo neomuscularization [94]. These structural abnormalities increase the PVR to ≥3 Woods units. The characteristic remodeling observed in Group 1 PH can arise under a multiplicity of conditions, and is thought to be caused by the combined presence of two or more initiating factors (‘hits’). These can be genetic or epigenetic, but may also be associated with disease (e.g., schistosomiasis, connective tissue disease), exposure to certain drugs or toxins, or an increase in pulmonary arterial pressure and flow associated with congenital heart disease with the presence of a left-to-right shunt [93,94].

The most common forms of PH, Groups 2 and 3, are caused by left-sided heart disease and lung disease/chronic hypoxia (CH), respectively. Group 4 PH is due to pulmonary artery obstruction (e.g., caused by chronic thromboembolic disease), and Group 5 PH is associated with unclear or multifactorial mechanisms, including complex congenital heart disease and hematological disorders. Remodeling (e.g., medial hypertrophy) also occurs in these forms of PH, but is less pronounced and does not produce plexiform lesions or laminar concentric intimal fibrosis [94]. Increased pulmonary artery constriction due to Rho kinase-mediated Ca^2+^ sensitization has also been shown to have a pivotal role in in raising PVR in an animal model of Group 3 PH [95], and may be a contributing factor in several forms of human PH [96,97].

### 4.2. Mechanisms of Pulmonary Vascular Remodeling in PH

Thickening of the pulmonary arterial wall due mainly to an increased content of PASMCs in the arterial media is a key aspect of remodeling, and is generally characterized by augmented proliferation and diminished apoptosis of PASMCs [98]. On the other hand, particularly in Group 1 PH, increased apoptosis of PAECs is thought to occur at the onset of remodeling and to contribute to its initiation [99], for example by causing the release of transforming growth factor beta 1 (TGFβ_1_) which enhances PASMC proliferation [100]. Subsequently, the emergence of an apoptosis-resistant PAEC clonal population promotes the excessive endothelial proliferation which helps to drive the formation of the distinctive intralumenal lesions which characterize this type of PH [100].

PA remodeling in Group 1 PH, the most intensively studied type of PH, is driven by a constellation of factors which are only partially understood and to some extent vary depending on the precise nature of stimuli initiating the development of the disease [101,102,103]. However, there is evidence that PA hypertrophy is preceded and stimulated by remodeling of the extracellular matrix (ECM) [104,105,106]. ECM remodeling is characterized by an increased content and cross-linking of collagen within the vascular wall [107,108]. The expression of other matrix components, including tenascin, fibronectin, osteopontin and elastin, also increases, and the internal elastic lamina becomes fragmented.

ECM remodeling in PH begins before the rise in PAP [109] and is thought to result from various factors, including PAEC dysfunction, endothelial-to- mesenchymal transition (EndoMT) [107,110], and inflammation associated with the recruitment of multiple types of immune cells to the PA adventitia. These stimuli promote ECM remodeling by causing an imbalance between the proteolytic enzymes (e.g., lysyl oxidase, serum elastases, certain matrix metalloproteinases; MMPs) and their inhibitors (e.g., tissue inhibitors of matrix metalloproteinases; TIMPs), which regulate ECM structure [102]. ECM remodeling also increases the expression of endothelin-1 (Et-1) and fetal growth factor, both of which foster PA cell proliferation [109], and also of interleukin-6 (IL-6), one of the cytokines crucial for causing the PA inflammation which is another pivotal factor contributing to PA vasculopathy in PH [111].

ECM remodeling also results in an increase in PA stiffness which contributes to right ventricular failure by accelerating pulse wave reflection [107]. PA stiffness has additional actions which act at an early stage in PH to drive remodeling [112]. One of these is to stimulate the transcriptional co-activators YAP/TAZ (yes-associated protein/transcriptional coactivator with PDZ-binding motif) in pulmonary arterial adventitial fibroblasts, PASMCs, and PAECs [104,109]. Acting through the upregulation of miR-130/301, YAP/TAZ promote ECM remodeling by increasing the production of fibrillar collagen isoforms, lysyl oxidase, and connective tissue growth factor (CTGF), thus creating a vicious cycle in which ECM remodeling and PA stiffening reinforce each other, at the same time promoting PA cell proliferation and inflammation. Another important result of ECM remodeling and arterial stiffening exerted via YAP/TAZ is to assist metabolic reprogramming of PA smooth muscle and endothelial cells, a process which enables cells to produce increased amounts of substrates for the anabolic pathways required to support cell proliferation [113]. Metabolic reprogramming involves an upregulation of aerobic glycolysis accompanied by a suppression of oxidative phosphorylation (the Warburg effect) and also increased provision of oxaloacetate and glutamate to the tricarboxylic cycle (anaplerosis). YAP/TAZ upregulation facilitates metabolic reprogramming by increasing the expression of glutaminase 1, lactate dehydrogenase A and pyruvate carboxylase, all of which mediate anaplerosis [104]. Several factors contributing to the increased aerobic glycolytic flux have been identified, including an imbalance between mitochondrial fission and fusion [114], upregulation of pyruvate dehydrogenase kinase induced by hypoxic-inducible factor 1 alpha (HIF1α) [115], and the increased activity of 6-phosphofructo-2-kinase/fructose-2,6-bisphosphatase 3 (PFKFB3) [116].

## 5. Hydrogen Sulfide and PH

In retrospect, the first hint that sulfide might be useful in treating PH came from a study by Fallon et al. [117], which showed that five days of administration of garlic to rats abolished HPV, thought to contribute to chronic hypoxic PH. Although the authors did not propose a role for sulfide in this effect, garlic is a rich source of sulfide in the form of compounds such as diallyl disulfide and trisulfide. [118].

Evidence that sulfide exerted a beneficial effect in animal models of PH was then initially reported by Chao-shu Tang, Jin-bao Du and colleagues in 2003 [5], and these laboratories have subsequently carried out much of the research in this area. As described below, their hypothesis that sulfide is involved in animal models of PH essentially rests on two types of observation. The first is that plasma and pulmonary levels of sulfide are diminished in PH, and the second is that experimental interventions designed to increase or decrease sulfide levels in the body suppress and exacerbate, respectively, PH and the abnormalities in PA phenotype it is associated with.

### 5.1. Evidence That the Plasma Concentration of Hydrogen Sulfide Is Decreased in Human Pulmonary Hypertension

As of yet the effects of sulfide-donating drugs on PH in humans have not been described, although Morris et al. (2013) [119] reported that seven days of garlic ingestion did not affect various indices of lung function or exercise performance in volunteers exercising to exhaustion under hypoxic conditions. Nevertheless, the results of several studies support the notion that, as in animals, PH in humans is accompanied by a fall in the plasma sulfide concentration. Sun et al. [120] measured the plasma levels of homocysteine, H_2_S, CSE and CBS in 158 children with congenital heart disease who had either normal (control group; mean PAP average = 14.4) or high (PH group; mean PAP average = 52.0) mean pulmonary pressures. They observed that, compared to the controls, those with PH demonstrated higher levels of homocysteine and lower levels of H_2_S in the plasma. Levels of CBS were similar in both groups, but those of CSE were lower in the PH group. They also did a subgroup analysis comparing the levels of these parameters in those with PH which was deemed to be either dynamic or obstructive (defined by a pulmonary vascular resistance of 3–10 and >10 Woods units, respectively). Compared to those with dynamic PH, plasma from the children with obstructive PH had more homocysteine, less H_2_S, similar levels of CBS, and less CSE. These results indicated that plasma levels of sulfide and homocysteine vary strongly and inversely over a wide range of PAPs, suggesting that they might be useful biomarkers for detecting the presence of PH. Unfortunately, however, these results can be questioned because of apparent mathematical discrepancies between the values of certain parameters (mean and systolic PAP, homocysteine and sulfide levels) shown in the tables setting forth the results of the main (PH vs. non-PH) as compared to the subgroup (dynamic vs. obstructive PH) analyses.

Tan et al. (2021) [121] compared plasma H_2_S and NO levels in age- and sex- matched children who were either healthy (controls), were about to undergo corrective surgery for congenital heart disease (pre-operative CHD), or had recently undergone corrective surgery for CHD (postoperative CHD). Children in the pre-operative CHD group were further divided into those who had a normal PAP, had mild PAH (systolic PAP = 31–45 mmHg), or had moderate-to-severe PAH (systolic PAP ≥ 46 mmHg). Those in the postoperative CHD group were divided into those with normal PAP and those with PH (systolic PAP > 30 mmHg) following surgery. It was found that both NO and H_2_S in the plasma were significantly lower in the pre-operative CHD groups with PH, compared to the controls. The extent of this decrease was proportional to the degree of PH. Moreover, plasma levels of H_2_S increased 24 and 48 h post-operation in patients who had a systolic PAP (sPAP) of <30 mmHg after the surgery, but remained lower in those in whom the PAP remained >30 mmHg. In addition, plasma H_2_S concentrations at 24 and 48 h after surgery were inversely correlated with VIS-max scores (a measure of the severity of disease) at these times. In contrast, although post-operative plasma NO levels were similarly correlated with the VIS-max, they were not affected by the sPAP after surgery. The authors speculated that the relationships between H_2_S, NO and sPAP that they had observed reflected the effect of CHD on pulmonary pressure, with higher pressures leading to endothelial dysfunction.

Chen et al. (2005) [122] reported that serum sulfide levels were significantly higher in patients with stable chronic obstructive pulmonary disease (COPD) compared to controls (healthy volunteers with no lung disease), but were similar to the controls in those with acute exacerbation of COPD (AECOPD). In line with the findings in patients with CHD, a subgroup analysis of patients with AECOPD showed that the serum H_2_S content was 27% lower in patients with a high systolic PAP (sPAP ≥ 35 mmHg) than in those with a sPAP in the normal range (<35 mmHg). In a subsequent investigation, Sun et al. (2015) [123] found that the sulfide content of lung tissue from smokers with COPD was similar to that of both smokers without lung disease and healthy non-smokers. On the other hand, the protein expression of CSE was higher in the non-smokers than in the other two groups (where it was similar), although mRNA for CSE was much higher in the smokers with COPD than in the other two groups.

Liao et al. 2021 [124] examined the relationship between the plasma sulfide concentration, expression of CSE and CBS, the main PA diameter, and sPAP in patients with COPD. They found that the plasma sulfide concentration was negatively correlated with the main PA diameter. Since the main PA diameter tends to increase in PH [125], this supports the idea that the PA is associated with a fall in plasma sulfide. On the other hand, they also found that the mRNA expression of CSE was positively correlated with the mean PAP and the sPAP, and that of CBS was also positively correlated with the sPAP, implying that the fall in plasma sulfide was associated with an increased expression of these sulfide synthesizing enzymes. This seems paradoxical, although, as described above, this laboratory detected reciprocal changes in CSE mRNA and protein expression in COPD patients [123].

Taken together, these results suggest that plasma sulfide levels are depressed in several types of human PH. However, more work needs to be carried out to firm up this possibility, especially because the levels of plasma sulfide observed in two of these studies [120,121] were much higher than those currently thought to exist (see Section 5.3). In addition, in each investigation, patients were suffering from other cardiac or pulmonary conditions which might themselves have affected sulfide metabolism.

### 5.2. Sulfide in Animal Models of PH

Numerous animal models have been developed in an effort to define the pathogenesis of PH and investigate possible treatments. The two most long-standing and widely used models involve exposing animals to chronic (i.e., several weeks of) hypoxia or administering an injection of monocrotaline (MC), a toxin which, after being converted in the liver to MC pyrrole, is thought to initiate the development of PH by damaging the pulmonary arterial endothelium [126,127,128]. Although both models were initially developed to study PAH (i.e., Group 1 PH), it was pointed out by Rai et al. 2008 [129] that neither exhibits the hallmark vascular lesions (plexiform lesions and laminar concentric intimal fibrosis) seen in human PAH. They also noted that PA vasoconstriction contributes more to the increase in PVR in these models than it does to human PAH, which in most patients does not respond to vasodilator treatment. Whereas the pathogenesis of the CH model in animals presumably resembles that of Group 3 PH in humans, the use of MC has been criticized because its effects on the lung do not reiterate those observed in any specific class of human PH [126]. However, it remains a valuable tool for characterizing certain aspects of PH in general (e.g., the roles of endothelial dysfunction and inflammation, the effectiveness of anti-remodeling drugs [130]). The realization that CH and MC used alone are of limited value for studying the pathogenesis of Group 1 PH, taken with the recent explosion of information about the role of genetics, molecular biology, gender and inflammation in its development [103], has led to the development of numerous new animal models of this condition, many of which employ two or more hits, in an effort to mimic its characteristic features more closely [130].

Notably, however, almost all of the studies evaluating the role of sulfide in PH in animals have used CH or monocrotaline. In most of the CH studies in rats and (and one in broilers [131]) animals were exposed to normobaric hypoxia (10% O_2_, 6 h/d) for 3 weeks. On the next day, following in vivo measurement of PAP, they were euthanized to allow harvesting of lung tissue and plasma. For all of the investigations with monocrotaline, Sprague Dawley rats were given an IP injection of MC (60 mg/kg body weight), and PH was then allowed to develop for 3 (or in one study 1 to 4 [132]) weeks before euthanization. The remainder of in vivo studies of sulfide employed a PH model in which an systemic-to-pulmonary (aortocaval) shunt was introduced to cause high pulmonary flow and pressure. This is a model for Group 1.4.4 PH, which is thought to be initiated by the combined effects of increased pulmonary artery pressure and shear stress on endothelial cells [94]; this situation arises in infants born with congenital heart malformations causing a significant pulmonary to systemic (left-to-right) shunt (e.g., a ventricular septal defect). Following shunting, animals were left for either 4 or 11 weeks before experiments were initiated.

The investigations into the role of sulfide in PH using these models generally incorporated the use of three experimental groups. These comprised 1. animals undergoing the PH-inducing intervention, 2. controls in which the PH-inducing intervention was replaced by a sham procedure, and 3. animals undergoing the PH-inducing intervention together with daily intraperitoneal (I.P.) injections of either sulfide (usually but not always in the form of NaHS) or of the irreversible CSE inhibitor proparglylglycine (PPG). Some of the studies included both NaHS and PPG groups, with the doses of NaHS or PPG sometimes varying between PH models and laboratories.

As shown in Table 1, Table 2, Table 3 and Table 4, these three models were found to share several important features with regard to the relationship between pulmonary sulfide levels and the development of PH (see tables for references). Firstly, the rise in PAP which occurred in each model was accompanied by decreases in the expression of CSE, the production of sulfide by lung tissue, and the plasma sulfide concentration. Secondly, administration of NaHS or other sulfide donors during the treatment used to induce PH diminished or reversed the decreases in CSE expression and plasma sulfide, as well as inhibiting the increase in PAP, and attenuating various changes in PA phenotype associated with PH. Thirdly, although the consequences of treatment with PPG were not characterized as thoroughly as those of applying a sulfide donor, PPG augmented the rise in PAP in all three models. In MC-treated animals PPG also exacerbated medial thickening of small PA, and in the shunt model it similarly worsened PA remodeling. Therefore, PPG, which enhanced the PH-associated fall in plasma, exaggerated the manifestations of PH, whereas treatment with a sulfide donor, which acted to maintain plasma (and lung tissue) sulfide levels, lessened the severity of PH. In addition, although a detailed histological characterization of the PA endothelium was not carried out in these studies, in each PH model the endothelium appeared to be abnormal, with PAEC hypertrophy, and irregularity of the internal elastic lamina [5,133,134]. In each case, these changes were reported to be lessened by NaHS treatment.

Notably, decreases in CSE expression and lung sulfide production similar to those observed in these three rat models of PH also occurred in a mouse model of COPD and accompanying PH evoked by daily exposure to cigarette smoke (CS) for 12 or 24 weeks [147]. CBS expression also fell. As in the rats, daily I.P. injection of NaHS during the COPD/PH induction protocol largely prevented the rise in right ventricular systolic pressure (a measure of PAP) and the development of pulmonary vascular remodeling. The fall in CSE/CBS expression and the decrease in the lung sulfide production rate also did not occur in the NaHS-treated mice.

In contrast to these results and the universal finding in rats that PH was accompanied by a fall in CSE expression, Rudyk et al., 2019 [29] reported that the expression of CSE, as well as NADPH oxidase 4 (Nox4) and SOD3, in PAs and lung tissue was increased in a chronic hypoxic model of PH in mice. CSE expression was similarly increased in lung tissue from humans with Group 1 PH. Paradoxically, however, the levels of CysSSH and GSSH and other sulfide species (HS^−^, HSS^−^, and thiosulfates), were decreased in mouse lungs and plasma after 14 days of hypoxia, although plasma levels of protein persulfides increased. They also demonstrated that either continual administration of PPG using an implanted minipump during the 14-day period of CH, or a single injection of anti-CSE siRNA, worsened right ventricular hypertrophy (RVH; an index of PH severity), whereas administration of a persulfide donor had the opposite effect. They proposed that CH was causing an increased production of lung H_2_O_2_ (via Nox4) and sulfide (via CSE),and that these were reacting to form per- and polysulfides which were persulfidating protein kinase Gα1 (PKGα1) causing its activation [59]. This would provide a countervailing vasodilating/anti-remodeling influence to limit the development of PH. In line with this hypothesis, levels of disulfide PKGα1 were increased in PAs and lung from PH mice, and administration of polysulfides using an osmotic mini-pump during the hypoxic run in period increased the proportion of the disulfide form of PKGα1 and ameliorated PH, as shown by a lowering of right ventricular pressure and RVH. On the other hand, administration of PPG or anti-CSE siRNA worsened PH and prevented the increase in PKGα1 disulfide induced by hypoxia. The importance of PKGα1 activation in countering the development of chronic hypoxic PH was further supported by the observation that PH was significantly worsened in Cys42Ser PKGIα knock-in (KI) mice in which PKGIα was resistant to oxidation.

Interestingly, the combination of decreased concentrations of CySSH and GSSH with increases in both the expression of CSE (and CBS) and the level of cell oxidation was also observed in lung fibroblasts and bronchial epithelial cells from patients with COPD, compared to those from healthy controls [148]. The authors pointed out that per-/polysulfides are powerful antioxidants and speculated that their decreased levels in COPD might be associated with the oxidative stress which characterizes this condition.

The observation that CSE expression was increased in mice with PH caused by 2 and 4 weeks of CH appears to be at variance with the decreased CSE expression generally seen in the three rat models of PH, and also in broilers with chronic hypoxic PH [5,22,131,133]. However, it is intriguing that whereas lung production of sulfide in the aortocaval shunt model was decreased 11 weeks after shunting, it was increased 4 weeks post-shunting, when PH had not yet developed [142] (Table 3). This led the authors to suggest that this early rise in endogenous lung sulfide production might be acting to protect against PH, an idea that was supported by their later finding that PH was present at 4 weeks if shunted rats were treated with PPG [141] (see Table 1) and is in accordance with the similar proposal by Rudyk and colleagues for chronic hypoxic PH in mice. In contrast, the time course of the fall in the expression of CSE appears to parallel that of the development of PH in rats following the injection of MC, with no effect on CSE levels or PAP at 1 week but decreased CSE expression and PH at 2 weeks and thereafter [132,149].

### 5.3. CSE Expression, Plasma Sulfide Levels, and Lung Tissue Sulfide Production in PH

Before considering the mechanisms by which sulfide has been shown to exert its beneficial actions in animal models of PH, a further discussion of the effects of PH on plasma and tissue sulfide levels, and on CSE expression, is warranted.

The measurements of sulfide in human plasma in the investigations of PH described above were made using silver/sulfide electrodes [120,122], or a commercial spectrophotometry-based kit which was not described [121]. Plasma sulfide levels in control groups were found to be 38, 12, and 53 × 10^−6^ mol/L ([122], [120] and [121], respectively). In rats, most of the measurements of the plasma sulfide concentrations employed an N, N-dimethyl-p-phenylenediamine /methylene blue-based assay. Using this approach, four studies [5,135,136,137] reported plasma sulfide concentrations of ~3 × 10^−4^ mol/L, with other investigations reporting lower values of 51 [22,139] and 30 × 10^−6^ mol/L [132]. Using a commercially available H_2_S-selective sensor, Feng et al. (2017) [133] and Zhang et al. (2019) [145] recorded plasma concentrations of free H_2_S of 14 and 12.7 × 10^−6^ mol/L, respectively.

The methylene blue assay has been strongly criticized in that it is subject to numerous artifacts [36,150], and was shown in one study to measure plasma turbidity rather than the sulfide concentration [151]. More accurate methods, e.g., monobromobimane-based assays, have yielded estimates of plasma sulfide levels which are much lower than those described in the both the human and animal PH studies. For example, Rajpal et al. (2018) [152] found that the concentrations of acid-labile, sulfane, and free sulfide in human plasma were ~0.8, 0.3, and 0.5 × 10^−6^ mol/L, respectively. Comparable values for free, sulfane and acid-labile and total sulfur have been reported by others [17,153,154,155,156,157] in mouse, rat and human plasma, although free sulfide levels in the low nM range have also been described [16,158,159].

As shown in Table 2, Table 3 and Table 4, sulfide production by lung tissue was also diminished in all three rat models of PH. Most of the investigations used assays based on those developed by Stipanuk & Beck [160] and Zhao et al. [161] to assess the production or content of sulfide in biological samples. Measurement of sulfide production by CSE and CBS was carried in tissue homogenates supplemented with supraphysiological concentrations of their substrate (cysteine) and cofactor (pyridoxal 5ʹ phosphate. Using this approach, rat and broiler lung homogenates were seen in multiple studies [5,22,131,137,139] to produce sulfide at a rate of ~0.27 nmols/mg tissue wet weight per minute in control lungs and roughly half that much in PH lungs. Using the same technique, other laboratories [143] reported similar sulfide production rates in lung from controls and MC-treated rats of ~2.3 and ~1.6 nmols/mg protein/min (approximately 0.35 and 0.24 nmols/mg wet weight assuming protein contributes 15% of tissue wet weight/min) [162] and of 0.24 and 0.17 nmols/mg protein/min (0.036 and 0.026 nmols/mg wet weight) in lung from control mice and those exposed to cigarette smoke to induce COPD and PH [147]. Notably, an assay using experiments carried out using the fluorescent probe HSip 1 reported a not-too-dissimilar rate of sulfide production by intact hepatocytes of 0.008–0.040 nmol/mg/min, which the authors suggested was probably an underestimate since some of the sulfide produced would be lost to mitochondrial consumption [163]. Thus, the sulfide production rates observed in lung homogenates in the PH investigations appear to be physiologically feasible.

A substantial number of investigations have found that PH is associated with a fall in lung sulfide content. However, the sulfide levels recorded in almost all cases were very high. Using a methylene blue-based assay designed to measure tissue sulfide content [164], Han et al. 2011 [147] reported a lung content of ~1.5 μmol/mg protein sulfide in mouse lungs, which was unchanged in their model of cigarette smoke-induced COPD and PH. This equates to ~200 × 10^−3^ moles/kg tissue wet weight, a value roughly 3 orders of magnitude higher than the total labile sulfur levels currently thought to exist in a range of tissue types [16,165]. Employing a different assay, the origin of which was not cited, Li and colleagues [134,140,141,142] reported even higher sulfide content of ~30 μmol/mg tissue wet weight in control lungs and ~20 μmol/mg in lungs of rats with PH caused by aortocaval shunting, which translates to 30 and 20 mole/kg. However, in a later paper, Feng et al. (2017) [133], using an H_2_S selective sensor to measure the [sulfide] in the supernatant after the centrifugation of lung homogenates, reported that the sulfide concentration was reduced from ~1 to ~0.45 nmol/mg protein in controls rats vs. those with MC-induced PH. Although it is not clear to what extent this assay would have picked up the sulfane and acid-labile sulfide pools in the lung tissue, these values are more in line with current thinking about physiological levels of sulfide in tissues. For example, the total sulfide content (free + bound sulfane + acid-labile) in endothelial cells was recently measured as ~0.45 nmol/mg protein, equivalent to a total labile sulfur concentration of ~70 μmol/kg wet weight [61].

In conclusion, given the very high and probably erroneous levels of plasma and tissue sulfide reported in nearly every investigation of PH in which they were measured, the observations that this condition is associated with a fall in plasma sulfide must be interpreted with caution. Nevertheless, Shen et al., 2011 [166], in a comparison of the plasma sulfide concentrations in mice detected by the methylene blue- and monobromobimane-based assays, found that even though the sulfide level measured using the former was ~25X higher that reported by the latter, both assays detected a decreased plasma sulfide concentration in CSE knockouts. Indeed, the effect observed using the monobromobimane-based approach was much larger (~80 vs. ~30%), suggesting that methylene blue-based assays might underestimate changes in sulfide levels. In addition, these findings are consistent with the PH-associated decreases in CSE expression and in the physiologically realistic rates of production of sulfide in lung tissue which were recorded in multiple studies. Also providing indirect support for the possibility that plasma sulfide levels are low in PH are the many observations that the plasma sulfide concentration is decreased in other cardiovascular diseases. For example, Rajpal et al. (2018) [152] found that both the acid labile and bound sulfane sulfur pools were significantly lower in a combined group of patients with coronary artery and peripheral artery disease. Interestingly, increased risk of coronary artery disease was associated with a single nucleotide polymorphism in the cystathionine-γ-lyase gene (1364 G > T). Total plasma sulfide was also found to be lower in patients with congestive heart failure in two studies [167,168]. Plasma free and sulfane sulfur pools have also been reported to be significantly reduced in patients with heart failure with preserved ejection fraction compared to controls [165], although this was not seen in congestive heart failure [155]. Plasma sulfide was also lower in hypertensive humans [169] and rats [170] compared to normotensive controls

The question of why pulmonary CSE expression is apparently diminished in PH in rats and broilers [5,22,131,133] seems never to have been investigated. However, a similar fall in CSE expression was observed in the left ventricle in mouse model of heart failure, and was attributed to an increase in oxidative stress [171]. Treating the mice with the sulfide donor sodium thiosulfate reversed both the oxidative stress and the fall in CSE expression, a finding which echoes the observation that NaHS treatment restored CSE expression in the lungs of chronic hypoxic [5] and MC-treated [133] rats. Another possibility emerges from observations that the transcription factor nuclear respiratory factor 2 (Nrf2), the expression of which is diminished in PH, both promotes the expression of CSE and is upregulated by sulfide [172] (see below). There is also evidence that miR-21, which is upregulated during the early stages of PH in several types of cells in PAs [109], depresses the expression of CSE in aortic smooth muscle cells, the kidney, and the placenta [173,174,175], and that sulfide downregulates miR-21 [176,177]. Both the mutually reinforcing interactions between Nrf 2 and CSE and the antagonistic interactions between CSE and miR-21 could provide potential explanations for the opposing effects of PH and sulfide treatment on CSE expression, although these possibilities remain to be investigated.

Another unanswered question which emerges from these observations is to what extent the downregulation of CSE and resulting deficit in lung sulfide production is a cause rather than a result of other abnormalities driving its pathogenesis. There appear to be no reports of PH or RVH occurring either in CSE knockout mice or animals in treated with PPG. This may mean that the loss of sulfide production by this enzyme is not *per se* sufficient to cause PH, or alternatively that PH occurred but was not detected. However, evidence that administration of PPG following aortocaval shunting caused a mild degree of PH after 4 weeks which did not occur after shunting alone [141], and that PPG exacerbated PH once it had developed in these animals and those exposed to CH [29,135,141], suggests that endogenous sulfide production by CSE does act as a brake on PH. It would be interesting to see to what extent CSE knockout or blockade could function as an effective 2nd hit in combination with another intervention (e.g., bone morphogenetic protein receptor type 2 (BMPR2) knockout, Sugen).

Finally, although the fall in lung sulfide in PH has generally been ascribed to the decrease in CSE expression which accompanies it, a recent study by Combi and colleagues shows that sulfide levels in cells can alternatively be diminished by an increase in sulfide catabolism by the SOU, and that this may play a crucial role in promoting inflammation [178]. These authors observed that lower levels of bioavailable sulfide were present in calcified compared to non-calcified human aortic valve tissue. This was apparently due to an enhanced metabolism rather than a decreased synthesis of sulfide, since the expression levels of the SOU enzymes SQR, ETHE1, rhodanese, and SUOXwere increased, whereas the expression of sulfide producing enzymes was unchanged (CBS) or raised (CSE). Cell persulfidation was not decreased along with the level in cellular sulfide, possibly because the SOU enzymes can act as transpersulfidases. The results described in the paper indicated that calcific aortic valve disease, which resembles PH in that it is an inflammatory condition associated with a higher expression of the cytokines interleukin-1β (IL-1β) and tumor necrosis factor-α (TNF- α), is driven by an increase in mitochondrial sulfide metabolism. Using an in vitro model of valve calcification, the authors demonstrated that a very low concentration (5 × 10^−9^ mol/L) of the mitochondrially targeted sulfide donor AP39 was more effective than a much higher concentration of the general sulfide donor NaHS (25 × 10^−6^ mol/L) in preventing calcification. AP39, which has been reported to exert a wide range of anti-inflammatory effects in a variety of contexts (Magierowska et al., 2022; Karaman et al., 2023; Stachowicz et al., 2024) [179,180,181], also strongly suppressed the associated increase in cytokines. Since the pro-inflammatory and -calcifying effect of the decrease in sulfide levels in this tissue was apparently not associated with a change in protein persulfidation, these results suggest that a fall in mitochondrial [sulfide] due to its increased metabolism by the SOU can promote inflammation by an alternative signaling mechanism, which might involve an inhibition of respiration (recall that sulfide feeds electrons into the ETC via SQR).

A coincident increase in the expression of both sulfide-synthesizing (CSE, MPST) and -catabolizing (SQR, rhodanese, SUOX) enzymes has also been observed in senescent compared to non-senescent RAW 264.7 murine macrophages (Kieronska-Rudek et al., 2024) [182], and AP39 has been shown to exert a senostatic effect in endothelial cells (Latorre et al., 2018) [183]. Thus, mitochondrial sulfide levels and/or the rate of sulfide turnover may regulate multiple aspects of cell phenotype. In this case, a more comprehensive characterization of the effects of PH on the expression in PA cells of the enzymes responsible for the synthesis and catabolism of sulfide, as well as measurements of protein persulfidation, is likely to provide new insights into the interactions between sulfide and PA cell phenotype in this disease. In addition, these findings suggest that the effects of mitochondrially-targeted sulfide donors on PH should be tested.

## 6. Mechanisms by Which Sulfide May Inhibit the Development of PH (See Also Table 2, Table 3 and Table 4 for Summaries)

### 6.1. Involvement of Sulfide in Extracellular Matrix Remodeling of PA

Two investigations examined the effects of sulfide supplementation on indices of ECM remodeling in PH in rats. Hongfang et al. (2006) [136] reported that PH due to CH was a characterized by increases in the expression of collagens I and III and elastin in small and medium-sized PA, as assessed using a semi-quantitative immunohistochemical analyses. In addition, a semiquantitative in situ hybridization analysis showed that CH was associated with increases in mRNA for procollagens I and III and matrix metalloproteinase-1 (MMP-1), transforming growth factor β_3_ (TGFβ_3_) and urotension II in PA. All of these alterations were strongly depressed in animals that had received a daily injection of 14 μmol/kg body weight NaHS during the hypoxic treatment. Li et al. (2008) [140] subsequently demonstrated similar indications of ECM remodeling in PH 11 weeks following aortocaval shunting. Levels of hydroxyproline (a marker of collagen), collagens I and III, MMP-13, TIMP1, CTGF, and Et-1, all shown to be upregulated in lung tissue in various models of PH [184,185,186,187,188,189,190,191] were elevated in PAs from the shunt group compared to controls, as was the MMP-13/TIMP-1 ratio. Each of these increases were significantly blunted in shunted animals receiving 56 μmol/kg/day NaHS. The authors proposed that the down-regulation of Et-1 and CTGF caused by sulfide supplementation might be responsible for its effect on ECM remodeling.

Rudyk et al. (2019) [29] showed that several indices of EndoMT (increased expression of α-smooth muscle actin, desmin, Twist1, phosphorylated vimentin in lung tissue), as well as PA muscularization, were increased by CH in Cys42Ser PKGIα knock-in (KI) mice but not in controls. Based on other results presented in this paper as described above, this suggests that upregulation of sulfide production by CSE may serve to ameliorate CH-induced PH by promoting PKGIα -mediated suppression of EndoMT, although this remains to be established.

Hongfang et al. (2006) [136] found that CH caused an increase in the expression of proliferative cell nuclear antigen (PCNA) [192] in small and medium-sized PAs which was decreased by ~60% in animals treated with NaHS. In the 11 week aortocaval shunt model of PH, treatment with NaHS similarly suppressed an increase in PCNA expression, and also depressed the activation (phosphorylation) of extracellular signal-related kinase (ERK) [134], which promotes hypoxia-induced proliferation and inhibition of apoptosis of SMC [193,194]. Interestingly, PH and the accompanying increases in PCNA and phospho-ERK were not present 4 weeks after shunting [141,142], but were observed at this time if shunted animals had been treated with PPG. Li et al. (2009) [141] subsequently reported that the proportion of PASMCs undergoing apoptosis was decreased to the same extent at 4 and 11 weeks after shunting. At both time points, expression of Fas and caspase-3 fell, whereas the expression of bcl-2 increased. Apoptosis was further depressed in the 4-week shunt group if animals were treated with PPG, and the effects on Fas, caspase-3 and bcl-2 were exaggerated. In contrast, treatment with NaHS partially reversed the fall in apoptosis and the changes in Fas, caspase-3 and bcl-2 in the 11-week shunt group. These observations suggested that sulfide produced endogenously by CSE was delaying the onset of PH in the aortocaval model by depressing PASMC proliferation but enhancing apoptosis, and also that sulfide supplementation was able to counter the effects of shunting on proliferation and apoptosis even after PH was established.

### 6.2. H_2_S and NFκB in PH

The nuclear factor kappa-light-chain-enhancer of activated B cells (NF-κB) family of inducible transcription factors is important in organizing cellular immune and stress responses following its activation by a wide range of stimuli acting through a variety of receptors (e.g., T-cell, B-cell, inflammatory cytokine, pattern recognition, and Toll-like receptors), and has been implicated in multiple processes contributing to inflammation and PA remodeling in PH in both humans and animals [195,196,197,198].

The NF-κB dimer is held in an inactive state in the cytoplasm by its binding to an inhibitory I-κB family protein. Stimulation of its cognate receptors activates NF-κB through a process beginning with the phosphorylation of I-κB kinase (a complex of four proteins also referred to as IKK) on its β-subunit (IKKβ). IKK then phosphorylates both I-κB, leading to its ubiquination, dissociation from NF-κB, and proteasomal degradation, and p65 (RelA), one of the NFκB subunits, stimulating its activity [199]. This allows activated NF-κB to translocate to the nucleus, where it induces the transcription of genes coding for proteins involved in inflammatory, immune, and proliferative responses. Thus, activation of NFκB manifests as an decrease in I-κB but an increase in the I-κB-p/I-κB ratio, as well as the nuclear translocation and DNA binding of the NFκB dimer and the increased synthesis of numerous cyto- and chemokines, as well as a host of other proteins involved in inflammation (see http://www.nf-kb.org/target/index.html, accessed 12 December 2024).

Feng et al. (2017) [133] showed that each of these measures of NF-κB activation was present in lung tissue from rats with MC-induced PH, compared to controls. Moreover these indices of NFκB activation were significantly less prominent in animals given daily injections of NaHS following MC treatment. Similarly, they found that a 6 h treatment of cultured human PAECs with MC pyrrole caused an activation of NFκB in these cells which was lessened by co-treatment with NaHS. As in other studies, MC pyrrole decreased CSE expression and cellular sulfide production. These results suggested that the activation of NFκB shown to occur in PH is, at least in part, due to a fall in PAEC sulfide production, and that sulfide supplementation might exert a beneficial in PH by reversing this effect.

How might cellular sulfide levels regulate NFκB? Du et al. [200] had previously presented evidence that H_2_S inhibits NFκB-dependent production of monocyte chemoattractant protein-1 (MCP-1) in macrophages by persulfidating NFκB p65 on cysteine 38. They also showed that H_2_S suppressed the phosphorylation and degradation of I-κB, suggesting that it might additionally be preventing the activation of NFκB by inhibiting IKK. This led Zhang and colleagues [145] to investigate whether these mechanisms might be responsible for the ability of H_2_S to diminish NFκB—mediated inflammation in MC-induced PH. They demonstrated that H_2_S depressed the activity of NFκB in human PAECs by persulfidating both p65, on cysteine 38, and IKK, on cysteine 179 of its IKKβ subunit. They also reported that the activities of IKK, p65, and therefore NFκB, were increased by CSE knockdown, suggesting that resting levels of sulfide production were exerting a brake on NFκB which would be released by PH-inducing stimuli such as MC, hypoxia and altered shear stress, which diminished CSE expression. In additional experiments, they injected rats intra-bronchially with adeno-associated virus 6 containing either wild-type IKKβ or C179S IKKβ, and confirmed that they were expressed in the pulmonary endothelium. The rats were treated with MC to induce PH, with or without co-administration of NaHS. NaHS treatment inhibited PH, as well as PA remodeling and the increased lung expression of intracellular adhesion molecule-1, in MC-treated animals expressing wild-type IKKβ. These effects of NaHS were much diminished in rats expressing C179S IKKβ. This supported the hypothesis that in the induction of PH by MC is strongly dependent on the activation of NFκB, and that the ability of sulfide supplementation to reverse MC-induced PH was due to its inhibition of IKKβ.

Activation of NFκB induced by TGFβ [201] plays an important role in causing PAEC EndoMT and therefore PH [196,202,203]. One mechanism by which it does so is by increasing the stability and hence the activity [204] of the transcriptional regulator Snail1 [205,206]. Zhang et al. (2019) [132] examined whether inhibition of the NFκB/Snail1 pathway contributed to the suppression of MC-induced PH by sulfide. They confirmed the presence of EndoMT in their model of MC-induced PH, as demonstrated by decreases and increases, respectively, in the expression of VE-cadherin (PAEC marker) and α-smooth muscle actin, in PA. Measures of NFκB activation and the expression of Snail1 in lung tissue also rose. These changes were accompanied by the typical pattern of PA remodeling and the falls in lung CSE expression and plasma sulfide concentration observed by others. Daily treatment with NaHS (1 mg/kg/day) initiated immediately following MC treatment diminished remodeling and the indices of EndoMC. Treatment with PPG (10 mg/kg/day) had the opposite effects, implying that endogenous sulfide production acts to oppose the development of EndoMC and PH caused by MC. In further experiments, they observed that treatment of human PAECs with TGFβ_1_ evoked similar changes in the expression of VE-cadherin, α-SMA, and Snail1 as well as NFκB activation and a change in ultrastructural phenotype to a more smooth muscle-like appearance. These effects of TGFβ_1_ were strongly suppressed in human PAECs in which CSE was overexpressed, but were then largely restored if these cells were treated with PPG. In a subsequent investigation [146], this laboratory administered rats on a basis with inhalable porous microspheres containing ACS14, an H_2_S-releasing aspirin derivative, beginning a week after MC injection. A parallel group of MC-injected animals was treated with intragastric sildenafil. The effects of ACS and sildenafil on PH and EndoMt in these animals, and on EndoMT in human PAECs, was assessed using an approach similar to that they had adopted in the previous study. It was found that the ACS-containing microspheres (ACSMs) had effects on PH and EndoMT similar to those of both sildenafil and NaHS. ACSM inhalation caused a increase in the concentration of sulfide in the lung which reached a maximum after 24 h and was sustained at that level for at least another day. In contrast, little change in sulfide levels occurred in the plasma, heart, liver or kidneys. ACSM concentrations of up to 10^−4^ mol/L applied to PAECs in culture had little effect on apoptosis and cell viability.

Although these papers together support the concept that sulfide exerts an inhibitory influence on NFκB which could contribute to its beneficial actions in experimental PH, contradictory effects of sulfide on NFκB have been reported in the wider literature [207]. In contrast to the inhibition of NFκB described above, Sen et al. (2012) [208] observed that Ser 38 persulfidation activated NF-κB in liver cells, and others have also reported that sulfide increases the activity of NFκB [209,210,211]. This implies that the effect of sulfide on NFκB activity depends on the biological context and experimental conditions. This may also apply more widely, as sulfide has been shown to have a range of anti- and proinflammatory actions [212,213,214]. It has recently been demonstrated, for example, that whereas endogenous sulfide production by CSE in macrophages increased the activation of NF-kappaB by lipopolysaccharide, application of exogenous sulfide in the form of GYY4137 had the opposite effect [215].

Intriguingly, NaHS has also been shown to exert a protective action in acute lung injury caused by hypobaric hypoxia (5 days at a simulated altitude of 6500 m) [216]. A proteomic analysis on lung tissue suggested that NaHS was acting to counter proinflammatory effects of hypoxia exerted through the stimulation of NFκB. NaHS was also seen to activate a nuclear erythroid-related factor 2/HIF1α/hemoxygenase-1 pathway to upregulate the expression of anti-apoptotic and antioxidative mechanisms.

### 6.3. H_2_S, Nrf2 and Hemoxygenase in PH

One of the hallmark effects of H_2_S is to promote the nuclear translocation of nuclear erythroid-related factor 2 (Nrf2), a transcription factor which plays a central role in coordinating cellular responses to oxidant and electrophilic stress. Under basal conditions, cytoplasmic concentrations of Nrf2 are very low because it is ubiquinated and subjected to proteasomal degradation due to its interaction with the E3 ligase complex (Keap1-Cul3-Rbx1). This process is interrupted by H_2_S persulfidation of Keap1 [172,217,218]. This allows Nrf2 to accumulate and move into the nucleus, where it controls the expression of >240 genes coding for proteins involved in cytoprotective mechanisms, for example upregulating glutathione S-transferase, peroxiredoxin 1, thioredoxin-1, and isocitrate dehydrogenase to limit oxidative stress, increasing the expression of NDUFA4, uncoupling protein 3, and nuclear respiratory factor-1, while downregulating COX4I1, to maintain mitochondrial function and biogenesis, and decreasing the production of pro-inflammatory cytokines (e.g., TGFβ_1_, IL-6, IL-1β) and the adhesion molecule VCAM-1 [219,220,221]. Interestingly, Nrf2 has also been shown to increase the expression of both CSE and CBS, which may also contribute to its antioxidant effect because these enzymes can give rise to cysteine, and therefore glutathione, through the reverse transulfuration of homocysteine [172,222].

Nrf2 also increases the expression of hemoxygenase-1 (HO-1), which metabolizes heme to form CO, iron and biliverdin, the latter of which is converted to bilirubin, a potent antioxidant. CO exerts anti-proliferative actions on SMC by activating guanylate cyclase [223,224,225,226] (albeit much less effectively than NO; [227]). Qingyou et al. (2004) [135] showed that chronic hypoxic PH was associated with increases in the protein and mRNA expression of HO-1 in PA. Accordingly, there was a rise in the plasma concentration of CO. The increases in HO-1 expression and plasma CO concentration were potentiated in animals treated with NaHS during the 3-week hypoxic CH protocol, and were reversed in animals treated with the CSE blocker PPG (30 mg/kg I.P.) during this period. These results led the authors to suggest that sulfide-induced upregulation of the HO-1/CO can act as a brake on the development of chronic hypoxic PH, potentially acting by causing vasodilation, decreased release of vasoconstricting autocoids, and/or inhibition of PASMC proliferation.

Qinyou and colleagues did not evaluate the involvement of Nrf2 in the sulfide-mediated increase in HO-1 expression or in its beneficial effects on PH. However, Han et al. (2011) [147] later reported that the protein expression of Nrf2, along with that of CSE and CBS, was diminished in lung tissue in their mouse model of COPD and PH induced by daily exposure to cigarette smoke (CS) for 12 and 24 weeks. The expression of phosphorylated (i.e., activated) AKT (protein kinase B) was also decreased. In accord with the downregulation of Nrf2, two markers of oxidative stress (the GSSG/GSH ratio and 8-hydroxyguanine content) were also increased, as was cell death evaluated by terminal deoxynucleotidyl transferase dUTP nick-end labeling (TUNEL) and the activity of caspase-3. All of these effects were abolished or strongly suppressed in mice treated with 50 μmole/kg/d with NaHS during the period of CS exposure. In further experiments, exposure of cultured bovine PAECs to CS for 20 min similarly increased the activity of caspase-3 and decreased the protein levels of Nrf2, CSE, and CBS. These effects were largely prevented if the cells were exposed to 5 × 10^−5^ mol/L NaHS prior to CS. Sulfide exposure also increased the level of phosphorylated Akt. Importantly, siRNA knockdown of Akt largely abolished the ability of sulfide to suppress the effects of CS. The authors concluded that CS was promoting COPD and PH by causing the downregulation of phosphorylated Akt, which was decreasing the activity of Nrf2 and therefore the expression of antioxidant proteins, including CSE and CBS, and that this effect of CS was countered by sulfide. They did not investigate the mechanism by which sulfide was stimulating Akt, but there is extensive evidence that sulfide activates phosphatidylinositol 3-kinase (PI3K), which phosphorylates and turns on Akt [228]. These results are in line with the finding that glycogen synthase kinase-3β (GSK-3β) inhibits the activity of Nrf2 by preventing it from entering the nucleus, and that this effect is blocked by phosphorylated Akt [229]. Thus, sulfide appears to promote the activity of Nrf2 through at least two mechanisms.

The notion that the activation of Nrf2 by sulfide is important in inhibiting the development of PH is further supported by evidence that the level of Nrf2 in PASMCs was decreased by a 24 h incubation under hypoxic conditions, and that this effect was largely absent in the presence of sulforaphane which increases the concentration of Nrf2 by stabilizing the Keap1/Nrf2 complex, thus reducing the concentration of free Keap1 available to promote Nrf2 degradation [230]. Sulforaphane also raises cellular sulfide concentrations by activating CBS and CSE [231], and it is possible that its effect on Nrf2 is via sulfide-induced persulfidation of Keap1 since this occurs at Cys151, which is required for preventing Keap1-mediated degradation of Nrf2 by sulforaphane [232].

### 6.4. Other Potential Beneficial Effects of Sulfide on PH Pathogenesis

Acting through multiple mechanisms, H_2_S additionally enhances the synthesis of NO [233,234], which, along with its vasodilating properties, has antiproliferative effects on SMC [235]. However, the available evidence, although admittedly scant, is mixed with regard to the possibility that H_2_S treatment might exert a beneficial effect on PH by increasing NO synthesis in the lung. Li and colleagues [134,142] reported that whereas the production of both H_2_S and NO production by the lung were increased 4 weeks after introduction of an aortocaval shunt, after 11 weeks, levels of H_2_S had fallen below the control level although those of NO remained elevated. Also, NO production was diminished at 4 weeks in shunted animals which were treated with NaHS, and was increased at 11 weeks in animals treated with PPG, leading the authors to speculate that sulfide might be suppressing rather than potentiating NO production during the development of PH. On the other hand, this laboratory ([236] subsequently reported that the eNOS antagonist L-N^G^-Nitro arginine methyl ester inhibited the vasorelaxation of PAs to exogenous H_2_S and that block of CSE caused a similar effect on the vasorelaxation evoked by the NO donor sodium nitroprusside, suggesting that the increase in CSE expression engendered by sulfide treatment should potentiate the vasorelaxing and antiproliferative effects of NO, thereby lowering PAP in animals with PH.

This concept receives some support from a paper by Turhan and colleagues [143] which examined the effect of sulfide supplementation on PA contractile function in PH. MC was used to induce PH, and Na_2_S (2.5 mg/kg/day I.P.) was used as the sulfide donor. It was found that MC treatment diminished both the contraction to high K^+^ (indicative of Ca^2+^ influx via voltage-gated Ca^2+^ channels) and the relaxation of the high K^+^ contraction by acetylcholine (which is mainly due to endothelial NO release) in the main PA, compared to controls. Both effects of MC were reversed in the MC + Na_2_S group. A similar pattern occurred in PAs pre-constricted with phenylephrine. They also found that L-cysteine (10^−4^ to 2 × 10^−3^ mol/L) induced a concentration-dependent relaxation of the phenylephrine contraction. This relaxation was depressed in the MC group at higher cysteine concentrations, and was replaced by a contraction at lower cysteine concentrations. These effects of MC were abolished in the MC + Na_2_S group. The authors speculated that the suppression of the responses to both constricting and dilating stimuli by MC treatment were due to functional and/or structural damage to PASMCs and PAECs, and that the reversal of the MC effect by Na_2_S supplementation reflected a protective effect of sulfide. They also suggested that the vasodilation to cysteine might be mediated by increased sulfide synthesis, possibly acting to stimulate the NO/cyclic GMP axis, and that MC was depressing the cysteine relaxation by inhibiting sulfide synthesis.

Pulmonary oxidative stress is thought to contribute to the pathophysiology of groups 1 and 3 PH [237,238]. Wei et al., 2008 [137] found that CH altered several indices of oxidative stress measured in lung tissues in a manner with its increase. GSSG content and malondialdehyde content increased and total antioxidant capacity fell, although the expression of SOD1 and 2 was unaffected. Animals injected with NaHS during exposure to hypoxia demonstrated smaller changes in GSSG and total antioxidant capacity, although whether sulfide was specifically depressing the effects of hypoxia on these parameters is unclear, as it was not tested on a normoxic control group. Wu et al. [138] also reported exposure of cultured PASMCs to hypoxia for 24 h caused increases in oxidative stress and the expression of Nox4, as well as cell proliferation, migratory capacity, and apoptosis (reflected by the augmented activities of caspases 3, 8 and 9). They additionally found that PA remodeling and PH induced by CH were associated with the increased expression of proteins involved in endoplasmic reticulum (ER) stress (ATF6, GRP78). All of these effects were depressed by the sulfide donor GYY4137, and the increase in ER stress was inhibited by treating cells with anti-Nox4 siRNA. They proposed that oxidative stress due to the CH-induced increase in Nox4 expression in PASMCs, which has been shown to occur in human PAH and several animal models of PH [239,240], causes ER stress, which promotes vascular remodeling by altering PASMC proliferation, migration and apoptosis. By suppressing the hypoxia-induced increase in Nox4 expression, sulfide would inhibit this process. This concept is supported by observations in multiple types of cells that sulfide diminishes ER stress [157,241,242,243,244,245] and the activity and/or expression of Nox4 [246,247,248,249,250]. Unfortunately, however, the impact of this study is undercut by a lack of clarity regarding certain aspects of the methods and the statistical significance of some of the results.

There is increasing evidence that pyroptosis, a type of programmed cell death, plays an important role in vascular inflammation and remodeling in PH [198,251,252]. Pyroptosis is mediated by a protein complex, the NLRP3 (NOD, LRR and pyrin domain-containing protein 3) inflammasome, the formation of which in immune (and endothelial) cells is triggered by the presence of diverse damage-associated signals, including reactive oxygen species, hypoxia and stimulation of Toll-like receptor 4. Once assembled, the NLRP3 inflammasome activates caspase-1. This in turn activates cytokines, including IL-18 and IL-1β, which plays an important role in PH. IL-1β causes cleavage of the protein Gasdermin-D, the N-terminal domain of which moves to the plasmalemma to form large pores which cause pyroptosis and cytokine release. This process is primed by activation of NFκB, which upregulates the expression of IL-1β and also NLRP3, the sensor component of the inflammasome. Although its effect on pyroptosis specifically during PH has not been examined, there is evidence that sulfide inhibits pyroptosis under a variety of circumstances [253], and that it may do so by inhibiting NFκB p65 via persulfidation of cysteine 38 [254] and/or by persulfidating caspase-1 [255]. Studies of the effects of sulfide on pyroptosis and other forms of programmed cell death in PH would be of great interest.

The role of microRNAs in the pathogenesis of PH has been a subject of recent interest [256,257,258]. There seems to be relatively little overlap between the large sets of miRNAs which have been linked to PH and sulfide, respectively [103,256,258,259]. However, the expression of miR-21, which has been shown to promote vascular remodeling in PH through multiple mechanisms, including the decreased expression of BMPR2 [109,260,261], is downregulated by sulfide in heart and lung tissue, in both cases resulting in a decrease in fibrosis [176,177]. Nevertheless, it is not known whether sulfide interacts pulmonary miR-21 in PH. Likewise, although both sulfide [262] and PH [263] have been linked to miR-214, whether or not the inhibition of miRNA-214 by sulfide observed in cardiac cells also occurs in PAs and has any implications for the pathogenesis of PH seems not to have been explored.

Another possible mechanism by which sulfide could ameliorate PH is the inhibition of metabolic reprogramming, which plays a crucial role in PA remodeling in PH. Although the effect of sulfide on metabolic reprogramming in PA cells has not been examined in PH, a study examining the pathogenesis of atrial fibrosis and fibrillation [242] demonstrated that, compared to patients in sinus rhythm, the left atrial appendage in patients with atrial fibrillation exhibited signs of the Warburg effect (increased glucose consumption, lactate content, expression of lactate dehydrogenase and pyruvate dehydrogenase kinase 4, decreased expression of pyruvate dehydrogenase). These were accompanied by a decreased sulfide content and expression of CSE and MST, with an increased expression of CBS. Similar changes in metabolism and the expression of these sulfide-synthetizing enzymes were observed in left atrial tissue from a rat model of angiotensin 2—induced atrial fibrosis compared to controls, but these changes were largely absent in angiotensin 2 -treated rats given 56 mg/kg/day NaHS. The effects exerted by NaHS on atrial fibrosis were equivalent to those seen in angiotensin 2- treated rats given dichloroacetate, a pyruvate dehydrogenase kinase antagonist which suppresses the Warburg effect and has been shown to ameliorate animal models of PH and idiopathic PAH in humans [264,265,266].

## 7. Summary and Conclusions

**Figure 2:**
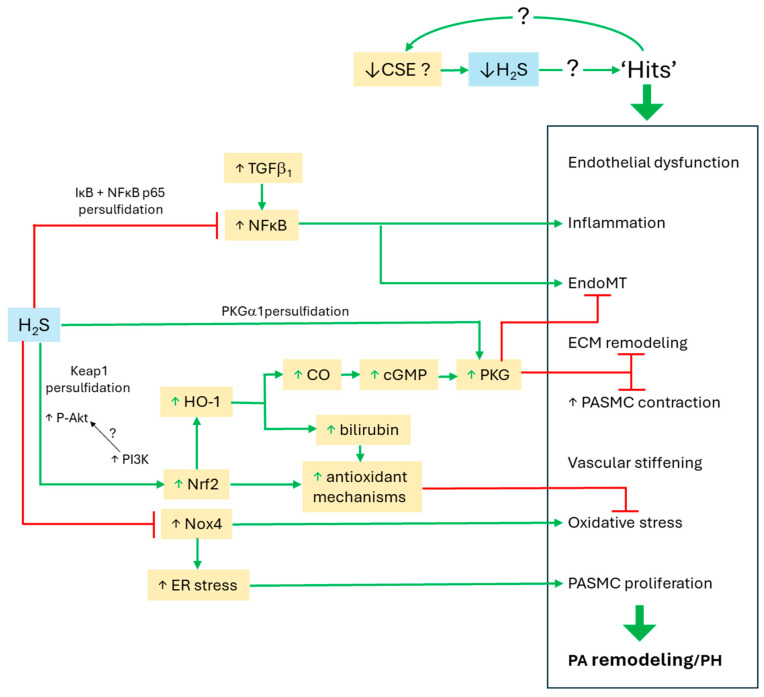
Mechanisms by which sulfide may inhibit pathological processes leading to PH. Green arrows between and within boxes indicate stimulation by sulfide. Red arrows represent inhibition by sulfide. Black arrows within boxes indicate increases in activity and/or expression associated with pulmonary hypertension. ↑ and ↓ represent increases and decreases respectively, in activity, concentration, or expression. Text adjacent to arrows shows putative mechanisms by which sulfide is exerting effects. Boxes at the top represent potential interactions between CSE, sulfide and ‘hits’ which initiate and/or sustain PH. It is possible (but not yet proven, as indicated by the question marks) that a fall in CSE expression (and/or other changes in sulfide metabolism not yet described) may contribute to the pathogenesis of PH, and/or that factors predisposing to PH are responsible for changes in sulfide metabolism. Mechanisms shown represent the present author’s interpretation of information presented in references [29,132,133,135,138,147].

To date more than two dozen papers focusing on the involvement and actions of hydrogen sulfide in PH have been published. The general consensus is that CSE expression, as well as plasma and lung levels of sulfide, are diminished in PH, and that sulfide supplementation mitigates several animal models of PH by suppressing a range of associated manifestations of PA remodeling. The best documented mechanisms by which sulfide is seen to lessen the severity of experimental PH are suppression of EndoMT and the activity of NFκB [29,132,133,145,146], stimulation of PKGα1 [29], activation of Nrf2 via AKT phosphorylation [147] and possibly a decrease in ER stress [138]. Although their relevance to models of PH other than those that they were seen in is unknown, Figure 2 is a speculative attempt to draw these mechanisms together. Evidence from the wider literature implies that sulfide could also be working through many additional mechanisms, including potentiation of the NO/sGC/cyclic GMP/PKG axis at other levels, suppression of oxidant stress, other anti-inflammatory actions, retardation of endothelial senescence [99,183] and reversal of metabolic reprogramming. Observations that H_2_S donors have pleotropic inhibitory actions on the processes responsible for PA remodeling support the notion that a sulfide -based treatment for PH is likely to be efficacious [267]. It is also clear that sulfide donors are beneficial in in vivo and in vitro models of other conditions such as diabetic cardiomyopathy, heart failure, atrial fibrillation, ischemia-reperfusion injury, and atherosclerosis [20] which share with PH important pathogenic features, including inflammation [268], oxidative stress [269], fibrosis [270], and mitochondrial dysfunction [271].

Small clinical trials exploring the use of sulfide donors in various cardiovascular diseases have been carried out or are in progress [20]. SG1002, a microcrystalline form of alpha sulfur (S8) containing trace amounts of polar molecules such as sodium sulfate and sodium polythionates bound on its surface, which render it water soluble [267], can be given orally. It has been shown to be beneficial in several animal models of heart failure/dysfunction [156,157,272,273,274] and has been administered to heart failure patients for 3 weeks in ascending doses (200–800 mg) to examine its effect on blood sulfide levels [155]. It has also been administered at a dose of 1500 mg/d for 75 days to subjects with idiopathic oligoasthenozoospermia [267]. In both studies it was well tolerated with no signs of toxicity. ATB-346, a sulfide-releasing form of naproxen, has been tested in phase 2 trials, first as a treatment for osteoarthritis [275] and then to determine if it caused less gastrointestinal ulceration than naproxen [159]. Over a 14-day treatment period, it caused a 94% smaller incidence of GI ulcers than did naproxen, and was shown to elicit a comparable increase in liver transaminase levels. In addition, the ACE inhibitor zofenopril and the anti-thrombotic agent clopidogrel have been shown to release sulfide [276,277]. Additional sulfide donors have been patented and are being developed [267]. Moreover, sulforaphane, which appears to act as a stimulus for endogenous sulfide production [231] has been administered to patients in numerous small clinical trials for a variety of conditions [278], and has been shown to suppress pulmonary vascular remodeling in chronic hypoxic PH [279] and to inhibit cardiac remodeling in the MC and Sugen 5416/hypoxia models of PH [280,281]. The beneficial effects on MC-induced PH of inhalable porous microspheres containing ACS14, which appears to selectively deliver sulfide to the lung and thereby potentially minimize toxicity elsewhere in the body [146], point to an alternative option for treatment which will hopefully be pursued further in future investigations. Evidence that the mitochondrially-targeted sulfide donor AP39 exerts anti-inflammatory actions in multiple contexts (see Section 5.3) suggests that its effectiveness in PH is also well worth testing, especially because evidence that it is effective at very low concentrations suggests that it might be able to exert a beneficial action without causing as many side effects as a non-targeted donor.

The availability of several sulfide donors which appear to be suitable for use in humans means that it should be feasible to use animal models to generate the information about dosing, efficacy, and toxicity, especially with extended treatment, which will be required before clinical trials can be contemplated. In particular, since in all of the studies reporting positive effects of sulfide donors on PH in animal models these agents have been administered during the PH-inducing protocol, an important issue which must be addressed in future investigations in animals is whether they can offer similar, and lasting, benefits when administration is initiated after PH is established. It is noteworthy, for example, that the upregulation of Nrf2, which is likely to be a key mechanism by which sulfide exerts its beneficial actions in PH, has been proposed to retard the initial development of cardiovascular and other diseases, but to become ineffective or indeed counterproductive once the disease is established [221]. In addition, whereas observations that sulfide supplementation was of benefit in four dissimilar animal models of PH are certainly promising, it has not been tested in any of the newer multiple-hit models, for example those designed to mimic deficient BMPR2 signaling. Importantly, there is also no information about the efficacy of sulfide supplementation as a treatment for PH in female animals. Nevertheless, given the remarkable advances in our understanding of the biology of sulfide and RSS over the past decade, the current availability of sulfide donors which should be able to produce stable and controllable levels of plasma sulfide, and the lack of truly effective treatments for Group 1 and 3 PH, it would appear that further and more substantial efforts to explore the use of various types of sulfide donors for treatment of PH are warranted.

## Figures and Tables

**Table 1:** Effects of sulfide treatment on mean pulmonary artery pressure (mPAP) and plasma [sulfide] in rat models of pulmonary hypertension (PH).

PH Model	mPAP Control mmHg	mPAP ^1^ PH mmHg	mPAP PH + H_2_S mmHg	mPAP PH + PPG mmHg	Plasma H_2_S Control μmol/L	Plasma H_2_S PH μmol/L	Plasma ^2^ H_2_S + PH + NaHS	Plasma ^3^ H_2_S + PH + PPG	Ref.
CH 3 weeks Wistar	16.3	23.7	16.3		301	192	317		[5]
	14.8	20.5	14.4	25.8	294	196	324	142	[135]
	15.7	23.7	16.3		299	195	271		[136]
	16.3	23.7	16.3		300	187	309		[137]
CH 4 weeks Sprague- Dawley	≈14 ^4^	≈34	≈26		≈152	≈96	≈130		[138]
CH 3 weeks broilers	≈23	≈37	≈25						[131]
AC shunt 11 weeks Sprague-Dawley (SD)	15.9	23.6			51	36			[22]
	15.8	23.7			51	36			[139]
	26.3	39.1	31.3						[134]
	17.3	27.6	24.2						[140]
	17.1	27.5	23.2						[141]
AC shunt 4 weeks SD	15.5	16.4	19.5						[142]
	15.5	16.2		20.3					[141]
MC 3 weeks Wistar	≈22	≈34	≈22						[143]
MC 3 weeks Wistar	≈15	≈51	≈37		≈14	≈9	≈12		[133]
MC 3 weeks Wistar	≈18	≈38	≈25						[144]
MC 3 weeks Wistar	≈17	≈34	≈25		≈12	≈4	≈14		[145]
MC 3 weeks SD	≈15	≈36	≈26	≈43	30	16			[132]
MC 3 weeks SD	≈13	≈38	≈22						[146]

Abbreviations: Propargylglycine (PPG); aortocaval (AC); monocrotaline (MC). ^1^ Systolic PAP and right ventricular pressure used instead of mean PAP in references [134,143], respectively. ^2^ and ^3^ animals given I.P. NaHS or PPG while undergoing CH treatment. ^4^ In this and other tables, all values shown as approximations (≈number) were derived from inspection of figures. Exact values are from tables.

**Table 2:** Effects of chronic hypoxia (CH) on cystathionine γ lyase (CSE) expression in lung tissue, and effects of treatment with sulfide and other agents on changes in pulmonary artery (PA) phenotype induced in chronic hypoxic rat models of pulmonary hypertension (PH).

PH Model	Effect of PH and Sulfide Treatment on CSE mRNA and/or Protein Expression in Lung Tissue.		Lung Sulfide Production nmole/mg Wet wt /min		Effects of Treatment with Sulfide, and Other Agents on Changes in PA Phenotype Induced by CH or Tobacco Smoke	Ref.
	PH vs. Control	PH + H_2_S Vs. PH	Control	PH		
CH 3 weeks Wistar rats	↓ mRNA, protein	↑ mRNA, protein	0.278	0.127		[5]
					CH ↑ plasma [CO] and PA expression of HO-1. Treatment with 14 mg/kg/day NaHS further ↑ [CO] and HO-1 expression.	[135]
					CH ↑ PA expression of urotensin 2, collagens 1 & 3, elastin, TGFβ_3_, PCNA, procollagens 1 & 3. Treating with 14 mg/kg/day NaHS ↓all of these effects.	[136]
			0.289	0.187	CH ↑ oxidized glutathione and malonaldehyde and ↓ total antioxidant capacity in lung tissue. 14 mg/kg/day NaHS treatment ↓ the effect of PH on total antioxidant capacity & oxidized glutathione	[137]
CH 4 weeks SD					CH ↑ expression of endoplasmic reticulum stress-related proteins ATF6 & GRP78. These effects were absent in rats treated with GYY4137 (dose not stated).	[138]
CH 3 weeks broilers	↓ mRNA, protein		≈0.28	≈0.12		[131]

Abbreviations: Carbon monoxide (CO); hemoxygenase-1 (HO-1); transforming growth factor beta 3 (TGFβ_3_); proliferating cell nuclear antigen (PCNA). The symbols ↑ and ↓ indicate an increase or a decrease, respectively.

**Table 3:** Effects of AC shunting on CSE expression in lung tissue, and effects of treatment with sulfide and other agents on changes in PA phenotype caused by the AC shunting model of PH in rats.

PH Model	Effect of PH on CSE mRNA and/or Protein Expression in Lung Tissue.	Lung Sulfide Production in nmol/(g wet wt/min) or Content in μmol/mg ^1^		Effects of Treatment with Sulfide, and Other Agents on Changes in PA Phenotype Induced by Aortocaval Shunting	Ref.
	PH Vs. Control	Control	PH		
AC shunt 11 weeks SD rats	↓ mRNA in PA, ↓ protein in lung tissue	0.26	0.13		[22]
	↓ mRNA in lung tissue	0.26	0.13	L-arginine (1 g/kg body weight) given daily following shunting prevented the ↑ in PAP, PA remodeling and ↓ in lung CSE expression and plasma [sulfide] induced by shunting.	[139]
		*30.2*	*20.2*	Shunting ↑ PCNA, phosphorylation of extracellular signal-related kinase (ERK), endothelial nitric oxide synthase (eNOS), and nitric oxide (NO) synthesis by lung tissue. NaHS 56 mg/kg/day ↓ these effects, also ↑ HO-1 expression and CO production in lung tissue.	[134]
		*30.2*	*20.2*	Shunting ↑ expression of collagens 1 and 3, matrix metalloproteinase-13, tissue inhibitor of metalloproteinase 1, connective tissue growth factor in intra-acinar PA. ↑ hydroxyproline content in lung tissue. ↑ plasma [endothelin-1] and endothelin-1 mRNA in lung tissue. NaHS 56 mg/kg/day ↓ all of these effects.	[140]
		*32.8*	*24.2*	Shunting ↓ proportion of apoptotic pulmonary artery smooth muscle cells in lung sections and ↓expression of Fas and caspase-3 and ↑ expression of bcl_2_. NaHS 56 mg/kg/day following shunting ↑ proportion of apoptotic cells, Fas and caspase 3 expression and ↓ bcl_2_ expression.	[141]
AC shunt 4 weeks SD rats		*14.4*	*37.6*	Shunting ↑ NO production and eNOS expression, and ↓ CO production and HO-1 expression in lung tissue. PPG mg/kg/day I.P.) following shunting exaggerated these effects and also ↑ PCNA expression & ERK phosphorylation.	[142]
		*23.6*	*32.7*	Shunting ↓ proportion of apoptotic PASMCs in lung sections and ↓ expression of Fas and caspase-3 and ↑ expression of bcl_2_. PPG (37.5 mg/kg/day injected I.P.) ↓ proportion of apoptotic PASMCs, Fas and caspase 3 expression, ↑ bcl_2_ expression.	[141]

^1^ Values in plain text refer to sulfide production in nmol/g wet weight/min and those in italics refer to sulfide content in μmol/mg. Note: The symbols ↑ and ↓ indicate an increase or a decrease, respectively.

**Table 4:** Effects of monocrotaline (MC) -induced PH on CSE expression in lung tissue, and effects of treatment with sulfide and other agents on changes in PA phenotype.

PH Model	Effect of PH and Sulfide Treatment on CSE mRNA and/or Protein Expression in Lung Tissue.		Lung Sulfide Production or Lung Sulfide Content		Effects of Treatment with Sulfide and Other Agents on Changes in PA Phenotype Induced by MC	Ref.
	Effect of PH vs. Control	H_2_S Effect vs. PH	Control	PH		
MC 3 weeks Wistar rats			2.28 nmol/mg/ protein/min	1.55 nmol/mg/ protein/min	MC ↓ vasoconstrictions to high K^+^ PSS and phenylephrine and also ↓ vasorelaxations to acetylcholine and cysteine. These effects were all greatly ↓ in rats treated with Na_2_S (2.5 mg/kg/day).	[143]
MC 3 weeks Wistar rats	↓ CSE protein in lung tissue	↑ CSE protein in lung tissue	≈1.0 μmol/g protein	≈0.45 μmol/g protein	MC ↑ expression of inflammation markers intercellular adhesion molecule-1 (ICAM-1), tumor necrosis factor α (TNFα), interleukins 6 and 8 (IL-6, IL-8), monocyte chemoattractant protein-1 (MCP-1) in plasma & lung tissue & activated activity of nuclear factor kappa-light-chain-enhancer of activated B cells (NFκB) in lung tissue. These effects were all greatly ↓in rats treated with NaHS (56 μmol/kg/day).	[133]
MC 3 weeks Wistar rats	↓ CSE protein and activity in lung tissue				MC↑ expression of ICAM-1, TNFα, IL-6 in lung tissue and plasma; also ↑ NFκB activity in lung tissue. These effects were ↓in rats treated with NaHS (56 μmol/kg/day) and in rats in which a persufidation-resistant variant of NFκB was expressed in PA.	[145]
MC 3 weeks SD rats	↓ CSE protein and activity in lung tissue				MC↓ expression of VE-cadherin and ↑ expression of a-smooth muscle actin, TGFβ_1_ and Snail-1 in PA; also ↑ activity of NFκB. These effects were ↓ in rats treated with NaHS (56 μmol/kg/day) and were ↑ in rats treated with PPG (10 mg/kg/day).	[132]
MC 3 weeks SD rats					MC↓ expression of VE-cadherin and ↑ expression of a-smooth muscle actin and Snail-1 in PA; also ↑ activity of NFκB. These effects were ↓ in rats treated with porous microspheres containing H_2_S-releasing drug ACS14.	[146]

Note: The symbols ↑ and ↓ indicate an increase or a decrease, respectively.
